# Functional Annotation of Genes Overlapping Copy Number Variants in Autistic Patients: Focus on Axon Pathfinding

**DOI:** 10.2174/138920210790886880

**Published:** 2010-04

**Authors:** Silvia Sbacchi, Francesco Acquadro, Ignazio Calò, Francesco Calì, Valentino Romano

**Affiliations:** 1 Dipartimento di Oncologia Sperimentale e Applicazioni Cliniche, Università degli Studi di Palermo, Palermo; 2 Molecular Cytogenetics Group, Centro Nacional de Investigaciones Oncologicas (C.N.I.O.), and Centro de Investigaciones de Enfermidades Raras (CIBERER), Madrid, Spain; 3 Associazione Oasi Maria SS. (I.R.C.C.S.), Troina (EN), Italy

**Keywords:** Autism Spectrum Disorders, Copy Number Variants, Gene Ontology, axon guidance signalling, neurodevelopment, candidate genes.

## Abstract

We have used Gene Ontology (GO) and pathway analyses to uncover the common functions associated to the genes overlapping Copy Number Variants (CNVs) in autistic patients. Our source of data were four published studies [1-4]. We first applied a two-step enrichment strategy for autism-specific genes. We fished out from the four mentioned studies a list of 2928 genes overall overlapping 328 CNVs in patients and we first selected a sub-group of 2044 genes after excluding those ones that are also involved in CNVs reported in the Database of Genomic Variants (enrichment step 1). We then selected from the step 1-enriched list a sub-group of 514 genes each of which was found to be deleted or duplicated in at least two patients (enrichment step 2). The number of statistically significant processes and pathways identified by the Database for Annotation, Visualization and Integrated Discovery and Ingenuity Pathways Analysis softwares with the step 2-enriched list was significantly higher compared to the step 1-enriched list. In addition, statistically significant GO terms, biofunctions and pathways related to nervous system development and function were exclusively identified by the step 2-enriched list of genes. Interestingly, 21 genes were associated to axon growth and pathfinding. The latter genes and other ones associated to nervous system in this study represent a new set of autism candidate genes deserving further investigation. In summary, our results suggest that the autism’s “connectivity genes” in some patients affect very early phases of neurodevelopment, i.e., earlier than synaptogenesis.

## INTRODUCTION

Autism Spectrum Disorders (ASDs) are a group of neurodevelopmental disorders that affects approximately 1 in 150 individuals and are characterized by deficits in reciprocal social interaction, communication and patterns of repetitive behaviours and restricted interests [5]. These “core deficits” can be variable among patients as far their severity and time of onset is concerned. In addition, non-core symptoms (mental retardation, epilepsy, gastro-intestinal disturbances, macrocephaly etc.), are observed only in subgroups of patients. This remarkable phenotypic complexity probably underlies multiple etiologies assumed to disrupt one or more phases of neurodevelopment.

A genetic etiology for ASD has been established and supported by several evidences including: (i) a higher concordance rate in monozygotic compared to dizygotic twins [6, 7], (ii) detection of chromosomal aberrations in up to 3% - 7% of children with autism [8], (iii) linkage and linkage disequilibrium studies, and (iv) a higher chance of association between autism with several monogenic disorders (all reviewed in [5]). These studies have also led to the identification of several loci implicated in ASDs etiology.

Despite these important progresses the genetic bases of Autism Spectrum Disorders remain poorly understood. A major factor responsible of this poor outcome relates to the complex genetic architecture of this disorder likely consisting of a marked multilocus and multiallelic heterogeneity with both common and rare risk alleles occurring in the autistic population [9]. Additional levels of genetic complexity would involve epigenetic and environmental factors, possibly interacting with risk alleles [10, 11].

In recent years, genomics has added a new dimension to autism’s research. Taking advantage of microarray technology, genomic analyses in autism have proceeded along three main directions: whole-genome expression profiling (e.g., [12]), whole-genome association studies (e.g., [13]) and the analysis of Copy Number Variants [1-3, 14-16]. These three experimental paradigms of genomic research have conceptual and technical differences, but on the same time they share the challenging task of extracting the autism-specific biological information from the large number of data typically generated in microarray analyses.

Copy Number Variants are operationally defined as submicroscopic insertions or deletions of DNA segments larger than 1 kb [17, 18]. These genomic mutations overlap thousands of genes, many of which play pivotal roles in biological pathways and may thus have profound effects on cell functions and human phenotype. In recent years there have been a growing number of studies demonstrating or suggesting that CNVs make a substantial contribution to human disease [19, 20]. The predicted functional effects of Copy Number Variants would include gene expression changes through a variety of mechanisms such as dosage effects, disruption of gene coding sequences, alteration of splicing, change in the location or efficiency of important regulatory elements, loss of heterozygosity, imprinting (all reviewed in [19]).

Further insight on the potential biological implications of CNVs has been gained by studying the common functions shared by genes overlapping CNVs detected in patients affected by various diseases using a variety of bioinformatic approaches. Relevant examples concerning diseases of the nervous system include amyotrophic lateral sclerosis (ALS), mental retardation (MR), schizophrenia (see Discussion for autism). For example, genes deleted specifically in patients with sporadic ALS encode proteins involved in oxidative phosphorylation, regulation of the actin cytoskeleton, and interactions between cytokines and their receptors [21]. Webber *et al.* [22] explored links between 148 MR–associated CNVs and phenotypes from 5000 mouse gene knock-out experiments and found that these CNVs were significantly enriched in those genes whose mouse orthologues, when disrupted, result in either abnormal axon or dopaminergic neuron morphologies. Genes disrupted by structural variants in patients with schizophrenia were significantly overrepresented in pathways important for brain development including neuregulin signalling, extracellular signal–regulated kinase/mitogen-activated protein kinase (ERK/MAPK) signalling, synaptic long-term potentiation, axonal guidance signalling, integrin signalling and glutamate receptor signalling [23].

Compelling evidences that CNVs are implicated in autism’s etiology relate to the observation that the frequency of *de novo* (rare) CNVs is significantly higher in cases versus controls [1, 2, 14]. On the other hand, the relative potential pathogenic role of both rare *de novo* and more common inherited CNVs is still under debate and several hypotheses invoking variable penetrance and expressivity, possibly influenced by other factors such as a mutation in modifier genes (not involved in a CNV), epigenetic mechanisms and/or environment have been proposed [9, 24]. Moreover, it is reasonable to assume that, in principle, only a subset of the tens or hundreds of genes (if not just one) overlapping a “pathogenic” CNV would actually be linked to autism.

In this study, we have used Gene Ontology and Ingenuity pathway analyses to uncover the common functions associated to deleted or duplicated genes in autistic patients. Using data from four published studies our functional annotation analysis uncovered several biological processes related to nervous system development and function potentially related to autism’s pathogenesis.

## METHODS

### Data Sets

The four CNV datasets we have used in this study refer to 4 distinct populations of autistic patients previously analyzed by microarray technology [1-4] accounting for a total number of 268 patients (see Supplementary Table **1** for details). Genes overlapping each CNV in patients were identified and gene symbols were manually retrieved using the information contained in the NCBI Map Viewer *Homo sapiens (human)* genome view Build 35.1. The symbols related to genes overlapping each CNV in each patient were grouped to generate several lists of genes named by the acronym “CS” (CS = Combined Set; see Table **1**). Each list was stored as a txt file and used for functional annotation analyses. An additional list of gene symbols corresponds to genes overlapping CNVs occurring in the general population that were downloaded from the Database of Genomic Variants (DGV: http://projects.tcag.ca/variation/). The version of DGV database (Build 35.1) used is updated on November 10^th^, 2008 and contains: (i) 31615 total entries (hg17), (ii) 19792 CNVs, (iii) a total of 6225 CNV loci. All data handling and storage were done using Excel (Microsoft).

### Cytoscape Networks

Bipartite networks were generated by Cytoscape software (http://www.cytoscape.org; version 2.6.2) with the aim of identifying the genes in CS1_All dataset that are shared by at least two patients. The input file contained (i) ID codes for the patients and (ii) the corresponding genes overlapping the CNVs in these patients. Two different types of nodes are displayed in the network (i.e., patient and gene). Nodes are connected only if a patient shares one or more genes with another or multiple patients. To increase the graphical resolution of the networks’ regions with high connectivity the “spring” layout was used.

### Gene Ontology and Tissue Expression Analysis

The function of genes overlapping CNVs in patients was annotated and analyzed according to the three organizing principles of Gene Ontology (GO: http://www.geneontology. org: BP: Biological Process, MF: Molecular Function, CC: Cellular Component). The Database for Annotation, Visualization and Integrated Discovery (D.A.V.I.D.) 2008_version 6^th^ (http://david.abcc.ncifcrf.gov) [25, 26] was used for GO analysis. For gene-enrichment analysis D.A.V.I.D. uses the “Ease score statistics”, an alternative name of Fisher Exact statistics, referring to the one-tail Fisher Exact test. P-values were corrected by the Benjamini correction which controls the False Discovery Rate (FDR). Lists not exceeding 2000 genes were uploaded to D.A.V.I.D. Separated analyses were carried out for CS “All”, “Gain” and “Loss” gene lists.

The “Tissue expression” function of D.A.V.I.D. was used to regionally map in nervous tissue the expression of genes from CS2_All dataset.

### Ingenuity Pathway Analysis

Ingenuity Pathways Analysis (Ingenuity Systems®, www.ingenuity.com) was used to identify the most significant biological functions, diseases and canonical pathways from the Ingenuity knowledge base associated to deleted/duplicated genes in autistic patients (CS1 and CS2 gene datasets). Separated IPA analyses were carried out for “All”, “Gain” and “Loss” gene lists. All IPA. analyses presented in this study were performed on august 2009. The significant over-represented functions, diseases and pathways were identified by the right-tailed Fisher's Exact Test using a threshold p-value < 0.05 after application of Benjamini-Hochberg method of multiple testing correction. Finally, network analysis was performed by IPA using each gene list. Briefly, IPA overlays test genes onto a global molecular network developed from information contained in the Ingenuity knowledge base. Networks of uploaded genes are then algorithmically generated based on their connectivity. The score assigned to each network is the negative log of the p-value, and is calculated by the right-tailed Fisher's Exact Test using the hypergeometric distribution. The threshold of p-value used was < 0.05.

## RESULTS

### Enrichment for Autism-Specific Genes

The actual data we retrieved from the four published studies are reported in Supplementary Table **1**. Specifically, the information on CNVs detected on autistic patients (size, start and end of each deletion or duplication), was used to manually retrieve altogether 2928 genes overlapping 328 CNVs. In these CNVs the number of genes ranged from 1 to 188. We named this gene dataset “CS0”. In order to generate a first set of autism-specific genes (enrichment step 1), we removed from “CS0” dataset, those genes which also overlap CNVs reported in Database of Genomic Variants. This new set, named “CS1_All”, contains 2044 genes overall duplicated or deleted in 233 patients. A second set of autism-specific genes (enrichment step 2) was obtained from CS1_All list by identifying those patients who share the same deleted or duplicated genes. To this end, we used Cytoscape to generate bipartite graphs for all genes from CS1_All dataset and related patients (see networks in Supplementary Fig. **1**). Thus, we overall identified 514 deleted or duplicated genes in 113 patients (the CS2_All gene list). The number of patients sharing at least one duplicated or deleted gene ranged from 2 to 10.

### Functional Annotation of Genes Overlapping CNVs

Gene Ontology analysis was carried out by the D.A.V.I.D. software with the aim of uncovering common functions associated to deleted or duplicated genes in autistic patients. The list of statistically significant (Benjamini corrected p-values < 0.05) GO terms obtained for each of the CS1 and CS2 gene lists is reported in Supplementary Table **2**. An inspection of this Table revealed two important features. First, a marked increase in number of GO terms obtained with CS2 gene lists compared to CS1 gene lists. Of the 90 statistically significant GO terms (all Benjamini corrected p values < 0.05) overall identified by D.A.V.I.D. with the 12 “CS1” and “CS2”gene lists, the highest number of GO terms (65 or 40 %) were detected only when the CS2 gene lists were used (see Table **2**). Table **2** clearly shows the strong effect generated by the two step enrichment strategy we have applied, i.e., the very low number of GO terms mapped by CS1 gene lists compared to CS2 gene lists. Consistent with the latter results, no statistically significant GO terms were obtained with CS0_Loss and CS0_Gain gene lists (containing the DGV genes). CS0_All could not be tested because it largely exceeded the maximum (i.e., 2000) number of genes that can be analyzed by D.A.V.I.D. We have also performed GO analysis on the excluded genes only (each All, Gain, Loss list was analyzed separately) and also in this case no statistically significant GO terms were obtained.

Second, nervous system development and function related genes were overrepresented in CS2 lists and particularly in the list of deleted genes (CS2_Loss). Totally, 20 genes from CS2 dataset were associated by D.A.V.I.D. to several nervous system-related GO terms. All 20 genes are associated to “nervous system development” GO term (see Table **3**). These 20 genes overlap 10 CNVs on 7 chromosomes, and they are deleted or duplicated in at least two autistic patients because belonging to CS2 list.

I.P.A. software was used to identify functions, diseases and canonical pathways associated to duplicated and deleted genes from autism-specific CS1 and CS2 gene lists, respectively (see Supplementary Table **3**). In Supplementary Table **3** only functions and diseases surviving the Benjamini-Hochberg multiple testing correction have been reported. Consistent with the results obtained with D.A.V.I.D., CS2 datasets could map many more functions than CS1 datasets. In addition, with only one exception (the “Parkinson’s signalling” canonical pathway, obtained with CS1_All gene lists) all other functions, diseases and canonical pathways related to nervous system were exclusively mapped by CS2 gene lists.

In Table **3** we report all nervous system-related GO terms, functions, canonical pathways and diseases identified by the D.A.V.I.D. and I.P.A. softwares respectively with their associated genes.

The “Network” function of I.P.A. was used to investigate known protein–protein and gene–gene interactions possibly existing among genes overlapping CNVs in autistic patients. Several networks with highly significant scores were obtained for each of the three CS2 gene lists (“all”, “gain”, “loss”) (not shown). One of the top-scoring networks obtained with CS2_All gene list is shown in Fig. (**1**).

### Genes Related to Axon Pathfinding

As it can be seen in Table **3**, among the various I.P.A. functions, there are several ones involved in processes related to axon pathfinding. From canonical pathway analysis performed with IPA using CS2_Loss list without applying the multiple testing correction, “axonal guidance signalling” also emerged as the most significant pathway (p = 0.00531 using the right-tailed Fisher's Exact Test) with 6 associated genes from the latter list. Based on these observations we then screened the longer CS1_All gene list to look for additional genes associated to “axonal guidance signalling” Canonical Pathway. This search led to identify additional 15 (21 in total) genes related to this pathway (also reported in Table **3**). The total number of patients bearing a deletion or a duplication involving one or more of these 21 genes is 23. Since the genes of the CS1_All list refer to 233 patients (see above) we would expect 10% of autistic patients (23/233) bearing a defect in this pathway. The position, role and interactions of proteins encoded by these genes in the “axonal guidance signalling” pathway, drawn using “Path designer” graphical tool of I.P.A. software, are shown in Supplementary Fig. (**1**).

### In Silico Tissue Expression Analysis

Using “Tissue expression” function of D.A.V.I.D. we have also mapped the regional expression of CS2_All genes within nervous system. On a total of 514 D.A.V.I.D. could only annotate 221 genes whose 139 (see Supplementary Table **4**) were expressed in nervous system and many of them appear to be expressed in several brain regions that have been shown to be altered in autism such as cerebral cortex, cerebellum, amygdala.

## DISCUSSION

In this study we have attempted a reconstruction of molecular and cellular mechanisms potentially involved in autism’s etiology by functionally annotating genes overlapping CNVs detected in patients. The source of CNVs data were four published CGH-microarray studies [1-4] altogether carried out on a large autistic population. Our meta-analysis was based on three assumptions: (i) the number of autism susceptibility genes overlapping CNVs detected on a single patient may be insufficient to recover significant information in functional annotation analyses, (ii) only a subset of all genes overlapping a “pathogenic” or “potentially pathogenic” CNV would be actually linked to autism, (iii) several duplicated or deleted genes from different CNVs and patients may participate to the same pathogenic mechanism (e.g., a pathway).

Starting from the original list of CNVs’ genes (CS0) we have applied a two-step strategy to generate two new lists (CS1 and CS2) assumed to be strongly enriched in autism-specific genes. In addition, the analyses were carried out separately for deleted or duplicated genes. This procedure was used with the aim of increasingthe signal-to-noise ratio in functional annotation analyses. Here, by “signal” we mean the number of putative autism susceptibility genes overlapping a CNV, and by “noise” all remaining genes in the same CNV or in CNVs that do not contain genes related to autism. It is worth noting here that the exclusion of deleted or duplicated genes in patients that are also reported in Database of Genomic Variants (enrichment step 1) may have also caused the removal of some susceptibility genes for autism. Indeed, such susceptibility genes are expected to occur in the general population and therefore are likely to be reported in DGV database which contains only data originally described in healthy controls. For instance, we noted that a strong candidate gene for autism, NRXN1 [2, 4, 15, 16, 27], did not survive the first filtering procedure and was not used for functional annotation analyses. Despite these considerations it is reasonable to expect that the number of susceptibility genes for autism would be higher in autistic patients than in non-autistic individuals implying that the number of excluded genes after the first enrichment step (CS0>CS1) was probably very low. Moreover, as our results show (summarized below) the exclusion of the DGV genes revealed to be a critical and necessary step to increase the sensitivity of our functional annotation analyses. Support to this prediction is provided in our results by at least four observations: (i) no detection of statistically significant GO terms with CS0 lists, (ii) no detection of statistically significant GO terms with the list of excluded genes, (iii) detection of a significantly higher number of GO terms, functions and pathways obtained with CS2 gene lists compared to CS1 gene lists, (despite the large difference in genes’ number between the two lists, see Table **2**), (iv) nervous system related genes are largely overrepresented in CS2 gene lists compared to CS1 gene lists (see Supplementary Tables **2**, **3**, **4**). Specifically, many more nervous system related functions, pathways and GO terms were detected by CS2_Loss (deleted) compared to CS2_Gain (duplicated) gene lists. This result is consistent with the observation that most CNV mutations in persons with autism are deletions [1]. Furthermore, it may also be speculated that the putative pathogenic role of some genes of CS2_Loss list would consist in an unmasking of a recessive mutation (loss of heterozygosity) caused by a *de novo* CNV loss in some patients.

We noted that seven genes in CS2_Loss list have been tentatively or strongly linked to autism in previous studies many of which did not use CNV analysis. A first group include WNT2: [28]; SEMA5A: [29]; CADPS2: [29-31]; CTTNBP2: [32]. In addition, the arginine-vasopressin (AVP) gene (deleted in one patient from the [1], dataset) may be considered a candidate gene for autism because of its functional partner. Indeed, the AVP gene encodes the ligand for the AVP receptor 1a (AVPR1a) and polymorphisms in this last gene have been shown to be in linkage and in linkage disequilibrium with autism [33]. Furthermore, two other deleted genes have also been previously reported in autistic patients, namely FOXP2 [34] and OXT [1]. It is worth mentioning here that FOXP2 protein dramatically down-regulates CNTNAP2, a strong candidate gene for autism [35, 36], whereas OXT gene encodes the neuropeptide binding the oxytocin receptor (OXTR), another strong candidate gene for autism [37-40].

Several studies have reported that in some autistic patients CNVs affect genes involved in synapse formation and function [2, 4, 41, 42]. In other studies that have used overrepresentation analyses, genes overlapping CNVs detected in autistic patients have been annotated to a variety of functional categories including those related to cell adhesion [15], ubiquitin pathway and neuronal cell-adhesion [16], glycobiology [43]; phosphatidylinositol signaling pathway [42]. It is interesting that these functional categories refer to very basic mechanisms that also play important roles in various phases of neurodevelopment such as those we have identified in our functional annotation analysis, i.e., neurogenesis, neuron migration, axon pathfinding. This suggests that a multiplicity of molecular functions potentially affected by CNVs may underly the same altered cellular process. Moreover, this pathogenic model is consistent with the genetic heterogeneity underlying autism because mutations in different genes from different patients would disrupt the same molecular pathway or cellular process [9]. In turn, a variety of molecular/cellular functions may also account for the phenotypic heterogeneity observed in autistic patients. Indeed, though the metapopulation of autistic patients we have investigated is homogeneous for autism diagnosis (same core symptoms), is expected to be heterogeneous for (i) different degree of severity of autistic core symptoms among patients, (ii) presence only in sub-groups of patients of peculiar clinical and sub-clinical phenotypes (beside the core symptoms). Inherently, patients from the four CNV studies [1-4] were not stratified according to specific endophenotypes.

Our functional annotation analysis has also uncovered several other biofunctions and pathways unrelated to nervous system (see Supplementary Tables **2** and **3**). For at least some of these functions e.g., those ones related to immune system and mitochondrion (just to mention few relevant examples), a strong link to autism has been proposed (e.g., [44, 45]). Alternatively such non-CNS related functions may be due in theory to other reasons. First, within a CNV, CNS related genes may co-exist with non-CNS related genes and if these genes share common non-CNS related functions they may be detected by IPA and GO (a sort of *hitchicking’s effect). *Second, th*e* same gene(s) may play multiple roles both CNS related and non-CNS related. Third, genes currently defined as non-CNS related may instead play a yet unknown role in CNS; these genes are currently classified in IPA and GO as non-CNS related terms, functions, pathways. Fourth, CNV in genes that are unrelated to autism are included in the analysis as an effect of the heterogeneity (phenotypic and genetic) of population, in that not all CNVs in the genome of an autistic patient are related to autism even after removal of DGV genes. If such CNVs are enriched in genes sharing a common function (unrelated to nervous system), such function will be detected by IPA and GO. For example, we noted that some GO terms or IPA biofunctions and pathways were detected just because members of certain multigene families were clustered within a single CNV in a single patient.

The identification of 21 genes associated to “axonal guidance signalling” pathway raises the possibility that in a subgroup of patients (these 21 genes are overall deleted or duplicated in 23 patients) autism could be caused by a specific dysfunction in this early process of neurodevelopment**. **This number probably underestimates the fraction of patients having a genomic/genetic defect affecting axon pathfinding since genes/proteins not classified in this process may be involved too. Few examples in our results include proteins involved in mitochondrial function, cytoskeletal proteins among others.

Axon formation, growth and guidance is very important for establishing the correct pattern of neuronal connectivity during development. An altered pattern of connectivity (“developmental disconnection”) has been proposed as a major etiological mechanism for autism [46, 47]. Axon guidance signalling, in particular, regulates the progression of growth cone, a specialized structure sitting at the tip of migrating axons, guiding the axon until it reaches its final destination where it forms a synaptic connection with the right partner(s). This is a highly regulated, multistep process involving many molecules acting as extracellular guidance cues, growth factors, plasma membrane-bound receptors, signal transducers, regulators of cytoskeleton organization, etc. [48]. Interestingly, the 21 proteins associated in our study to “axon guidance signalling” appear to function at different levels of distinct but partly overlapping signalling cascades in this pathway (see Supplementary Fig. **2**).

Very recently published studies carried out on autistic patients have identified several genes (cited hereinafter) involved in axon pathfinding. The value of these data is enhanced by the diversification of methods and approaches used: APC [49] association study, [50]; PI3 kinases [51] review article, [42] CGH-microarray; Tsc1/2 [52]; SLIT1 [53] association study; ROBO3 and ROBO4 [54] association study; ROBO1 and ROBO2 [54] analysis of mRNA levels in lymphocytes; SEMA5A [29] analysis of mRNA levels in lymphoblastoid cell lines and [55] whole-genome association study; RUNX3 and SEMA4C [56] analysis of mRNA levels in peripheral blood cells; ADORA2A, GNAS, MARCKS, MYH10, SLIT2, EXT1, NFKB1, OMG, RHOA, DST, FNBP1 [12] analysis of mRNA levels in lymphoblastoid cells. Very recently, Glessner *et al.* [16] reported on the association of some genes overlapping CNVs in autistic patients with “axonal guidance signalling” pathway. It is intriguing that with very few exceptions (SEMA5A and some members of the ROBO family) in each of these studies, including our own, a different set of axon growth and guidance genes has been implicated. Thus our results and data from the literature provide overwhelming evidences in favour of axon pathfinding hypothesis proposed here, at the same time they well illustrate the recently proposed concept of “Autism: many genes, common pathways” [9].

Imaging studies have revealed functional anatomy alterations of distributed networks, as well as structural deficits of discrete white matter tracts in these patients [57-60]. These data, together with studies showing altered white matter maturation in autism [61] led to the development of the so-called “underconnectivity theory” of autism, which points to the interaction of multiple partial cortico-cortical and cortico-striatal disconnections as one of the main pathological processes of autism [58]. All these pathological and physiological findings in autistic patients might be explained also assuming deficits in the axon pathfinding process, in turn leading to an altered pattern of connectivity.

## CONCLUDING REMARKS

GO and IPA analyses have provided several lines of evidences that our two-step enrichment strategy has generated a set of genes (CS2 = 514 genes) many of which are probably related to nervous system development and function thus suggesting their relation to autism’s etiology. We have also investigated on the known expression of these genes in human tissues and found that many transcripts have been detected in nervous system with a significant proportion of them expressed in brain regions that have been found altered in autistic patients (e.g., amygdala, cerebral cortex, cerebellum, see Supplementary Table **4**). The precise mechanism by which these genes would interfere with neurodevelopment thus predisposing to autism may be different from gene to gene. Genes/proteins do not have only one function, the same gene can express a number of proteins and even a single protein can have multiple functions depending on the developmental stage, CNS area, protein levels, etc. Many genes annotated in our study to nervous system development and functions (see Supplementary Table **5**) have multiple functions suggesting that a variety of pathogenic mechanisms are implicated in autism.

The results of our study are also consistent with the hypothesis that the “connectivity genes”, in autistic patients, may affect several early phases of neurodevelopment thus providing further support to the etiological theory of “developmental disconnection” of autism [46, 47]. In our study we have in particular highlighted the potential pathogenic role of axon pathfinding. We propose that the majority of genes associated to nervous system development and function in this study represent novel candidates susceptibility genes for autism deserving further investigation.

## SUPPLEMENTARY MATERIAL

Supplementary material is available on the publishers Web site along with the published article.

## Figures and Tables

**Fig. (1):**
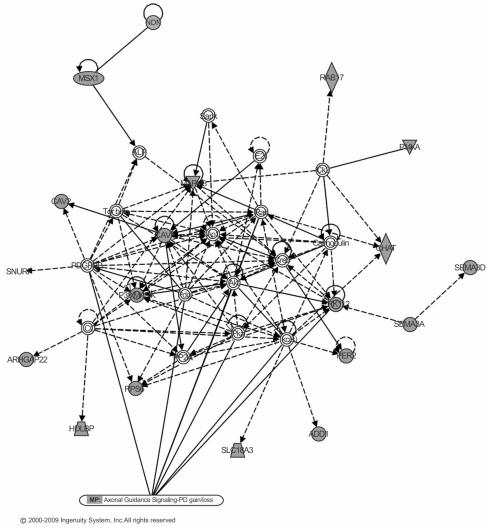
The figure shows one of several top-scoring multigene network associated by the Ingenuity Pathway Analysis software to seventeen genes (gray shaded symbols) of the CS2_All data set which includes both duplicated and deleted genes. Several genes associated to the axon guidance signalling canonical pathway and to other nervous system functions and diseases map in this network (see also Supplementary Fig. 2). A score of 29 (- log (p-value)) was obtained for this specific network calculated by the right-tailed Fisher's Exact Test.

**Table 1:** Description of the Gene Lists Used in this Study

*CS0*	The complete list of genes overlapping all CNVs detected in autistic patients from the four studies (cited in Supplementary Table **1**). The CS0 data set was not used for functional annotation analysis.
*CS1*	The list of genes obtained after subtracting from the CS0 list the same genes also overlapping the CNVs reported in the Database of Genomic Variants (http://projects.tcag.ca/variation/). The three gene lists from the CS1 dataset generated and used for functional annotation analysis are: (i) CS1_All (all genes), (ii) CS1_Gain (the duplicated genes), (iii) CS1_Loss (the deleted genes).
*CS2*	A sublist of the CS1 dataset referring only to the genes that are deleted or duplicated in at least two patients. The three gene lists from the CS2 dataset generated and used for functional annotation analysis are: (i) CS2_All (all genes), (ii) CS2_Gain (the duplicated genes), (iii) CS2_Loss (the deleted genes).

**Table 2:** Number of Genes in Each Dataset and Corresponding Number of Detected GO Terms

Dataset*	# Genes	# GO Terms**
CS1_ All	2044	23 (1%)
CS1_Loss	1074	32 (3%)
CS1_Gain	1158	7 (<1%)
CS2_All	514	65 (13%)
CS2_Loss	152	60 (40%)
CS2_Gain	254	2 (1%)

* See Table **1** for explanation of acronyms referring to data sets used.

** All GO terms are statistically significant (P-value corrected by the Benjamini method  < 0.05).

**Table 3:** Genes in CNVs of Autistic Patients Annotated to Nervous System Development, Functions and Diseases

GO Terms (D.A.V.I.D.)	Genes*
GO:0048699-generation of neurons	**SPON2**, ASZ1, WNT2, FARP2, SEMA5A, **LOC440258**, CAV2, **NDN**, PTPRZ1, SEMA3A, **CHAT**, CTTNBP2, CFTR
GO:0001764-neuron migration	ASZ1, WNT2, CAV2, **NDN**, CTTNBP2, CFTR,
GO:0007399-nervous system development	**SPON2**, SEMA3D, ASZ1, HES6, WNT2, FARP2, **C4orf6**, **LOC440258**, SEMA5A, CAV2, **NDN**, PTPRZ1, SEMA3A, DGCR14, FOXP2, **CHAT**, **MSX1**, CTTNBP2, **CRMP1**, CFTR
GO:0022008-neurogenesis	**SPON2**, ASZ1, WNT2, FARP2, SEMA5A, **LOC440258**, CAV2, **NDN**, PTPRZ1, SEMA3A, **CHAT**, CTTNBP2, CFTR
GO:0019905-syntaxin binding	ASZ1, WNT2, CAV2, CTTNBP2, CFTR
**I.PA. Functional Annotations**	
contact repulsion of axons	SEMA3A, SEMA3D
circadian rhythm of mice	CADPS2, PER2
collapse of growth cone	SEMA3A, SEMA3D, SEMA5A
chemotaxis of central nervous system cells	CAV1, SEMA3A
chemorepulsion of sympathetic neuron	SEMA3A
development of accessory nerve	SEMA3D
development of facial nerve	SEMA3D
development of glossopharyngeal nerve	SEMA3D
development of vagus nerve	SEMA3D
developmental verbal dyspraxia	FOXP2
familial advanced sleep phase syndrome	PER2
growth of retinal axons	SEMA5A
guidance of olfactory glomeruli	SEMA3A
innervation of first molar	SEMA3A
morphogenesis of sympathetic trunk	SEMA3A
pathfinding of retinal axons	SEMA5A
quantity of olfactory glomeruli	SEMA3A
Parkinson's Signaling	SEPT5, **CASP3**, GPR37, **MAPK8**, MAPK12, MAPK11
Axonal guidance signalling**	ADAM10, **BMP15**, **CRKL**, **EPHA7**, **FZD6**, MAPK12, MLC1, OPN1SW, PDGFA, **PIK3C3**, PLXNB2, PRKAR1B, **RAC3**, SEMA3A; SEMA3D, SEMA5A, SEMA7A, SMO, WASL, WNT16, WNT2

* Gene symbols in bold characters: duplicated genes, in plain text: deleted genes.

** This is the only case where the multiple testing correction was not applied (see text for further explanation).

## ABBREVIATIONS

ASD: = Autism Spectrum Disorders
CGH: = Comparative Genomic Hybridization
CNV: = Copy Number Variant
DAVID: = Database for Annotation, Visualization and Integrated Discovery
FDR: = False Discovery Rate
GO: = Gene Ontology
IPA: = Ingenuity Pathway Analysis
