# Supplementary material for: Functional Annotation of Genes Overlapping Copy Number Variants in Autistic Patients: Focus on Axon Pathfinding
Source: Curr Genomics. 2010 Apr;11(2):136–45. doi: 10.2174/138920210790886880 (PMC2874223; doi:10.2174/138920210790886880)
Supplement: Supplementary file 1 — Supplementary material is available on the publishers Web site along with the published article. [file CG-11-136_SD1.pdf]

SUPPLEMENTARY MATERIAL

Supplementary Table 1. It Contains Information on the Source of CNV Data Used in this Study, i.e. the Studies Published by [1-4]

Marshall *et al*., 2008: Data used are from Supplementary material, Table **S3**. "Table of All Autism-Specific CNV (NCBI build 35)".

Christian *et al*., 2009: Data used are from Spplementary material, Table **S2**: "AGRE aCGH abnormal results".

Sebat *et al*., 2008: Data used are from Supplementary material, Table **S3**. "Genes located within de novo copy number changes".

*Satzmari *et al*., 2007: Data selected from this study refer to validated CNVs only, detected in autistic patients (listed hereinafter).

SCAN ID	Family ID	Invd ID	chr	cytoband	start	stop	size (Kb)	cnv (loss/gain)	Validation
NAAR074-A7-04C35667	5116	5	1	1p31.3	60.389.500	61,350,000	960,5	gain	Familial
NAAR074-D1-04C35701	5116	4	1	1p31.3	60.629.100	61,350,000	720,9	gain	Familial, Affy Nsp 250K
NAAR007-D5-8172-201	8172	201	1	1q21.1	143.780.000	144,894,000	1,114,000	gain	Familial
NAAR007-D6-8172-202	8172	202	1	1q21.1	143.780.000	144,894,000	1,114,000	gain	Familial
NAAR070-D8-HI3079	1425	301	1	1q21.1	143.780.000	144,894,000	1,114,000	loss	Mendelian Error
NAAR032-G1-3222.004	3222	4	2	2p24.1	21.249.500	22.833.700	1,584,200	gain	Familial
NAAR022-E8-3020.004	3020	4	2	2q13	111.333.000	112,712,000	1,379,000	gain	Familial
NAAR006-A3-8125-201	8125	201	2	2q32.1	184.270.000	186,428,000	2,158,000	gain	Familial
NAAR006-A4-8125-202	8125	202	2	2q32.1	184.623.000	186,428,000	1,805,000	gain	Familial
NAAR007-B6-8159-203	8159	203	3	3p14.1	67.773.300	68,967,900	1,194,600	loss	Familial, Mendelian Error
NAAR037-A9-1014-A9-P3	3192	4	3	3p12.2	82.496.200	83,190,200	694	gain	Familial, Mendelian Error
NAAR052-F6-HI0682	1092	4	4	4p16.3\t4p16.2\t4	398,952	17,044,800	16,645,848	loss	Mendelian Error
NAAR062-H3-HI1083	1218	3	4	4p16.3\t4p16.2\t4	398,952	6,790,030	6,391,078	gain	Mendelian Error
NAAR052-F6-HI0682	1092	4	4	4p15.32\t4p15.31	18,188,800	23,734,300	5,545,500	loss	Mendelian Error
NAAR010-F7-8268-201	8268	201	4	4q26	117,347,000	118,226,000	879	gain	Familial
NAAR047-F8-01C08500	7126	4	4	4q35.1\t4q35.2	185.809.000	189.310.000	3,501,000	gain	Familial
NAAR062-D6-HI0890	1088	4	4	4q35.2	188,901,000	189,905,000	1,004,000	gain	Familial
NAAR062-D7-HI0891	1088	5	4	4q35.2	188,901,000	189,310,000	409	loss	Familial
NAAR023-F9-3046.003	3046	3	4	4q35.2	189.670.000	190.118.000	448	loss	Familial, Mendelian Error
NAAR073-E12-04C35554	5035	3	4	4q35.2	189,670,000	190,118,000	448	gain	Familial
NAAR073-F2-04C35556	5035	4	4	4q35.2	189,670,000	190.200.000	530	gain	Familial
NAAR024-A12-3072.008	3072	8	4	4q35.2	189,808,000	191,091,000	1,283,000	loss	Familial, Mendelian Error
NAAR041-G7-01C05468	7008	4	4	4q35.2	190,013,000	191,091,000	1,078,000	loss	Familial, Mendelian Error
NAAR003-A5-8047-202	8047	202	5	5p15.33\t5p15.32	1,677,350	8,438,300	6,760,950	loss	Familial, Mendelian Error
NAAR047-E4-01C08484	7113	4	5	5q23.1	117,078,000	119,691,000	2,613,000	loss	Mendelian Error

(Suppl Table 1). Contd…..

SCAN ID	Family ID	Invd ID	chr	cytoband	start	stop	size (Kb)	cnv (loss/gain)	Validation
NAAR055-A4-HI0442	1066	4	6	6p25.2\t6p25.1\t6	3,801,430	18,594,500	14,793,070	loss	Mendelian Error
NAAR078-A3-200305415	4075	100	6	6q21\t6q22.1\t6	113,107,000	121,399,000	8,292,000	loss	Mendelian Error
NAAR052-G9-HI1097	1047	4	6	6q26	163,090,000	163,493,000	403	gain	Familial
NAAR063-F4-HI1524	1226	6	7	7p12.3\t7p12.2	49,519,500	50,046,900	527,4	gain	Familial
NAAR064-A4-HI1686	1245	9	7	7q21.11	83,174,700	85,227,400	2,052,700	loss	Familial, Mendelian Error
NAAR061-A5-HI0128	1010	5	7	7q31.32	121,543,000	122,291,000	748	loss	Familial, Mendelian Error
NAAR067-A8-HI2741	1261	8	8	8p23.2	3,909,530	3,909,710	1	gain	Familial
NAAR007-E6-8175-201	8175	201	8	8p23.1	8,160,870	11,422,100	3,261,230	loss	Mendelian Error
NAAR041-G2-00C04611	2029	3	8	8p22	13,570,600	14,168,200	597,6	gain	Familial
NAAR050-H5-HI2533	1356	302	9	9p24.3	239,391	559,992	320,601	gain	Familial
NAAR064-F6-HI2067	1305	3	9	9p24.3\t9p24.2\t9	239,391	33,916,500	33,677,109	gain	Familial
NAAR022-H7-3019.003	3019	3	9	9q21.12\t9q21.11	70,619,600	70,908,800	289,2	loss	Familial
NAAR065-F2-HI0182	1129	4	10	10p13	13,087,500	13,302,400	214,9	loss	Familial, Mendelian Error
NAAR060-A7-HI2165	1335	3	10	10q11.22\t10q11	49,217,200	50,638,800	1,421,600	gain	Familial
NAAR030-G10-3194.003	3194	3	10	10q21.3	68,424,900	68,552,100	127,2	gain	Familial
NAAR068-E11-HI2859	1440	303	11	11p11.12	49,879,900	50,595,000	715,1	gain	Familial
NAAR056-C2-HI0799	1203	4	11	11p11.12	50,096,900	50,595,000	498,1	gain	Familial
NAAR068-F2-HI2862	1440	302	11	11p11.12	50,096,900	50,595,000	498,1	gain	Familial
NAAR074-C12-04C35700	5081	4	11	11p11.12	51,247,900	51,319,500	71,6	gain	Affy Nsp 250K
NAAR001-F11-8017-202	8017	202	11	11q14.1	77,603,900	77,613,800	9,9	loss	Mendelian Error
NAAR063-B3-HI1190	1117	5	11	11q22.3	103,928,000	104,733,000	805	gain	Familial
NAAR041-F10-00C04574	9032	4	13	13q12.12	22,502,100	23,692,300	1,190,200	gain	Familia
NAAR054-F3-HI0298	1154	3	13	13q14.2	47,048,100	47,569,100	521	gain	Familial
NAAR054-F4-HI0299	1154	4	13	13q14.2	47,048,100	47,569,100	521	gain	Familial
NAAR031-G7-3212.003	3212	3	13	13q21.31\t13q21	63,529,600	65,319,400	1,789,800	gain	Familial
NAAR044-C5-01C06331	7084	4	13	13q21.32	65,120,700	65,951,300	830,6	gain	Familial
NAAR024-B7-3064.003	3064	3	15	15q11.2\t15q12	21,490,300	25,698,400	4,208,100	gain	Familial, Affy Nsp 250K
NAAR024-C7-3064.004	3064	4	15	15q11.2\t15q12	21,490,300	25,698,400	4,208,100	gain	Familial, Affy Nsp 250K
NAAR065-E6-HI0302	1097	3	15	15q11.2\t15q12	21,490,300	25,698,400	4,208,100	gain	Familial, FISH, MLPA
NAAR065-E7-HI0304	1097	4	15	15q11.2\t15q12	21,490,300	25,698,400	4,208,100	gain	Familial, FISH, MLPA
NAAR026-C4-3099.007	3099	7	16	16q21	60,141,700	61,581,600	1,439,900	loss	Familial, Mendelian Error
NAAR026-D4-3099.008	3099	8	16	16q21	60,141,700	61,581,600	1,439,900	loss	Familial, Mendelian Error
NAAR068-B12-HI1404	1263	5	17	17p12	14,304,400	15,237,700	933,3	gain	Familial

(Suppl Table 1). Contd…..

SCAN ID	Family ID	Invd ID	chr	cytoband	start	stop	size (Kb)	cnv (loss/gain)	Validation
NAAR001-E1-8012-201	8012	201	17	17p12	14,432,900	15,237,700	804,8	loss	Familial, Mendelian Error
NAAR022-A10-3023.004	3023	4	18	18p11.21\t18p11	10,741,600	11,601,400	859,8	gain	Familial
NAAR022-H9-3023.003	3023	3	18	18p11.21\t18p11	10,741,600	11,601,400	859,8	gain	Familial
NAAR036-B11-1014-B11-P2	3121	4	18	18q12.2\t18q12.3	34,993,000	35,619,700	626,7	loss	Familial, Mendelian Error
NAAR041-G7-01C05468	7008	4	20	20p13	95,685	691,22	595,535	gain	Familial
NAAR017-C2-HI2202	1318	3	21	21q22.11	30,538,900	30,983,100	444,2	gain	Familial
NAAR063-B1-HI1183_2	1004	4	22	22q11.21	17,506,900	20,306,800	2,799,900	gain	Familial, FISH
NAAR053-F1-03C15328	2072	2	22	22q13.33	48,004,600	48,881,300	876,7	loss	Familial, Mendelian Error, Affy Nsp 250K
NAAR072-F11-200501053	2072	1	22	22q13.33	48,004,600	48,881,300	876,7	loss	Familial, Affy Nsp 250K
NAAR078-A3-200305415	4075	100	6	6q22.31\t6q22.32	122.787.000	130.388.000	7.601.000	loss	Familial
NAAR047-F9-01C08501	7126	3	4	4q35.1\t4q35.2	185.809.000	189.310.000	3.501.000	gain	Familial

*Four patients from this study were excluded because of the large no. of genes overlapping CNVs (NAAR052-F3-HI0633, CNV loss, 30,068,543 Kb, 386 genes; NAAR052-F6-HI0682, CNV gain, 86,168,200 Kb, 833 genes; NAAR078-A3-200305415, CNV gain, 50,824,300 Kb, 505 genes); columns' headings in this Table are as in Satzmari *et al*., 2007.

Supplementary Table 2. The Results of Gene Ontology Analysis Performed by the D.A.V.I.D. Software (http://david.abcc.ncicrf.gov) on the Genes Overlapping CNVs in Autistic Patients

Lists of genes*

	CS1_All		CS1_Gain		CS1_Loss		CS2_All		CS2_Gain		CS2_Loss	
GO Terms	# genes	p-value**	# genes	p-value**	# genes	p-value**	# genes	p-value**	# genes	p-value**	# genes	p-value**
GO:0005925 focal adhesion	-	-	-	-	-	-	5	1,07E-04	-	-	5	4,68E-06
GO:0045178 basal part of cell	-	-	-	-	-	-	5	1,49E-05	-	-	5	6,22E-07
GO:0005924 cell-substrate adherens junction	-	-	-	-	-	-	5	1,80E-04	-	-	5	7,96E-06
GO:0045121 lipid raft	-	-	-	-	-	-	6	6,29E-05	-	-	6	5,81E-04
GO:0005901 caveola	7	4,90E-05	-	-	6	4,04E-05	6	3,50E-07	-	-	6	1,30E-06
GO:0009925 basal plasma membrane	-	-	-	-	-	-	5	1,16E-05	-	-	5	5,79E-05
GO:0016599 caveolar membrane	7	1,47E-05	-	-	6	1,50E-05	6	1,25E-07	-	-	6	6,39E-09
GO:0030055 cell-matrix junction	-	-	-	-	-	-	5	2,53E-04	-	-	5	4,85E-07
GO:0030054 cell junction	-	-	-	-	-	-	12	6,51E-04	-	-	8	8,66E-04
GO:0044433 cytoplasmic vesicle part	-	-	-	-	-	-	8	7,32E-05	-	-	6	9,67E-05
GO:0030659 cytoplasmic vesicle membrane	-	-	-	-	-	-	8	5,25E-05	-	-	6	7,55E-05

(Suppl Table 2). Contd…..

	CS1_All		CS1_Gain		CS1_Loss		CS2_All		CS2_Gain		CS2_Loss	
**GO Terms**	**# genes**	**p-value****	**# genes**	**p-value****	**# genes**	**p-value****	**# genes**	**p-value****	**# genes**	**p-value****	**# genes**	**p-value****
GO:0019861 flagellum	-	-	-	-	-	-	5	2,82E-04	-	-	5	1,27E-05
GO:0012506 vesicle membrane	-	-	-	-	-	-	8	1,05E-04	-	-	6	1,27E-04
GO:0016310 phosphory-lation	-	-	-	-	-	-	20	3,98E-04	-	-	-	-
GO:0030154 cell differ-entiation	-	-	-	-	-	-	37	7,66E-06	-	-	-	-
GO:0006821 chloride transport	-	-	-	-	8	2,26E-04	8	4,70E-07	-	-	6	1,73E-06
GO:0042311 vasodila-tion	-	-	-	-	6	8,85E-05	6	8,72E-07	-	-	6	1,14E-08
GO:0051707 response to other organism	-	-	-	-	-	-	10	1,05E-04	-	-	-	-
GO:0046785 microtu-bule polymerization	-	-	-	-	5	6,07E-05	5	1,41E-06	-	-	5	4,34E-08
GO:0031116 positive regulation of microtu-bule polymerization	5	8,14E-05	-	-	5	1,07E-05	5	2,40E-07	-	-	5	7,30E-09
GO:0048609 reproduc-tive process in a mul-ticellular organism	-	-	-	-	-	-	7	5,31E-05	-	-	6	8,79E-06
GO:0031113 regulation of microtubule polym-erization	-	-	-	-	5	1,07E-05	5	4,76E-07	-	-	5	1,46E-08
GO:0031112 positive regulation of microtu-bule polymerization or depolymerization	-	-	-	-	5	2,10E-05	5	4,76E-07	-	-	5	7,30E-09
GO:0031110 regulation of microtubule polym-erization or depolymeri-zation	-	-	-	-	6	2,10E-05	5	3,71E-05	-	-	5	1,20E-06
GO:0051674 localiza-tion of cell	-	-	-	-	-	-	16	8,24E-06	-	-	-	-
GO:0031109 microtu-bule polymerization or depolymerization	-	-	-	-	6	2,24E-04	5	6,44E-05	-	-	5	2,11E-06
GO:0050880 regulation of blood vessel size	-	-	-	-	9	5,82E-06	6	4,06E-05	-	-	6	5,84E-07
GO:0022414 reproduc-tive process	-	-	-	-	-	-	12	1,25E-04	-	-	-	-
GO:0048545 response to steroid hormone stimu-lus	10	1,15E-05	-	-	9	7,35E-05	6	2,16E-05	-	-	6	3,04E-07
GO:0022008 neurogene-sis	-	-	-	-	-	-	13	3,29E-05	-	-	9	2,71E-05

(Suppl Table 2). Contd…..

	CS1_All		CS1_Gain		CS1_Loss		CS2_All		CS2_Gain		CS2_Loss	
**GO Terms**	**# genes**	**p-value****	**# genes**	**p-value****	**# genes**	**p-value****	**# genes**	**p-value****	**# genes**	**p-value****	**# genes**	**p-value****
GO:0048523 negative regulation of cellular process	74	1,48E-05	-	-	51	1,87E-05	30	4,13E-07	-	-	-	-
GO:0048519 negative regulation of biological process	80	4,18E-06	-	-	55	3,01E-06	32	8,72E-08	-	-	-	-
GO:0042127 regulation of cell proliferation	40	5,41E-05	-	-	31	3,73E-06	15	2,17E-04	-	-	-	-
GO:0008285 negative regulation of cell prolif-eration	26	1,48E-05	-	-	19	2,23E-05	15	5,35E-08	-	-	-	-
GO:0009615 response to virus	16	8,93E-06	14	6,64E-08	11	1,29E-04	10	3,27E-07	-	-	-	-
GO:0048468 cell devel-opment	-	-	-	-	-	-	28	2,44E-05	-	-	-	-
GO:0043627 response to estrogen stimulus	-	-	-	-	7	9,79E-06	5	4,50E-05	-	-	5	1,46E-06
GO:0007595 actation	-	-	-	-	-	-	5	5,41E-05	-	-	5	1,76E-06
GO:0035295 tube de-velopment	-	-	-	-	13	2,20E-04	8	4,32E-04	-	-	7	2,27E-05
GO:0035239 tube morphogenesis	-	-	-	-	-	-	8	6,37E-05	-	-	7	3,86E-06
GO:0048869 cellular developmental process	-	-	-	-	-	-	37	7,66E-06	-	-	-	-
GO:0001937 negative regulation of endothelial cell proliferation	6	6,39E-05	-	-	6	5,30E-06	6	4,73E-08	-	-	6	5,95E-10
GO:0001936 regulation of endothelial cell pro-liferation	-	-	-	-	6	3,75E-05	6	3,56E-07	-	-	6	4,58E-09
GO:0001935 endothelial cell proliferation	-	-	-	-	6	6,77E-05	6	6,59E-07	-	-	6	8,55E-09
GO:0003018 vascular process in circulatory system	-	-	-	-	9	7,01E-06	6	4,57E-05	-	-	6	6,59E-07
GO:0043434 response to peptide hormone stimu-lus	-	-	-	-	6	1,45E-04	5	4,50E-05	-	-	5	1,46E-06
GO:0042493 response to drug	-	-	-	-	-	-	6	2,28E-04	-	-	6	3,55E-06
GO:0030321 transepi-thelial chloride transport	-	-	-	-	5	2,10E-05	5	4,76E-07	-	-	5	1,46E-08
GO:0035150 regulation of tube size	-	-	-	-	9	5,82E-06	6	4,06E-05	-	-	6	5,84E-07
GO:0030317 sperm motility	-	-	-	-	-	-	5	3,71E-05	-	-	5	1,20E-06

(Suppl Table 2). Contd…..

	CS1_All		CS1_Gain		CS1_Loss		CS2_All		CS2_Gain		CS2_Loss	
**GO Terms**	**# genes**	**p-value****	**# genes**	**p-value****	**# genes**	**p-value****	**# genes**	**p-value****	**# genes**	**p-value****	**# genes**	**p-value****
GO:0051130 positive regulation of cellular component organization and biogenesis	-	-	-	-	6	1,89E-05	5	8,83E-06	-	-	5	2,78E-07
GO:0000003 reproduction	-	-	-	-	-	-	16	1,52E-04	-	-	-	-
GO:0006928 cell motility	-	-	-	-	-	-	16	8,24E-06	-	-	-	-
GO:0048699 generation of neurons	-	-	-	-	-	-	13	1,55E-05	-	-	9	1,56E-05
GO:0001764 neuron migration	-	-	-	-	-	-	6	7,83E-05	-	-	5	3,60E-05
GO:0016477 cell migration	-	-	-	-	-	-	11	2,13E-04	-	-	-	-
GO:0007399 nervous system development	-	-	-	-	-	-	20	1,03E-04	-	-	12	1,89E-04
GO:0032504 multicellular organism reproduction	-	-	-	-	-	-	7	5,31E-05	-	-	6	8,79E-06
GO:0032502 developmental process	-	-	-	-	-	-	52	3,86E-05	-	-	-	-
GO:0005132 interferon-alpha/beta receptor binding	7	5,58E-07	7	8,00E-09	6	1,26E-06	6	9,32E-09	-	-	-	-
GO:0000149 SNARE binding	-	-	-	-	-	-	5	8,23E-05	-	-	5	3,10E-06
GO:0050998 nitric-oxide synthase binding	-	-	-	-	5	8,22E-06	5	1,61E-07	-	-	5	5,61E-09
GO:0005515 protein binding	331	6,58E-06	-	-	203	1,52E-05	84	8,88E-05	-	-	-	-
GO:0019905 syntaxin binding	-	-	-	-	-	-	5	3,68E-05	-	-	5	1,36E-06
GO:0019900 kinase binding	-	-	-	-	-	-	8	2,21E-05	-	-	-	-
GO:0031098 stress-activated protein kinase signaling pathway	-	-	-	-	-	-	-	-	5	2,33E-05	-	-
GO:0032501 multicellular organismal process	-	-	-	-	-	-	-	-	23	6,05E-06	-	-
GO:0045177 apical part of cell	-	-	-	-	-	-	-	-	-	-	5	5,81E-04
GO:0005912 adherens junction	-	-	-	-	-	-	-	-	-	-	5	5,79E-05
GO:0005739 mitochondrion	-	-	-	-	-	-	-	-	-	-	13	3,02E-04

(Suppl Table 2). Contd…..

	CS1_All		CS1_Gain		CS1_Loss		CS2_All		CS2_Gain		CS2_Loss	
**GO Terms**	**# genes**	**p-value****	**# genes**	**p-value****	**# genes**	**p-value****	**# genes**	**p-value****	**# genes**	**p-value****	**# genes**	**p-value****
GO:0016324 apical plasma membrane	-	-	-	-	-	-	-	-	-	-	5	2,32E-04
GO:0016323 basolateral plasma membrane	-	-	-	-	-	-	-	-	-	-	5	4,03E-04
GO:0030324 lung development	-	-	-	-	8	1,76E-04	-	-	-	-	5	4,24E-05
GO:0051128 regulation of cellular component organization and biogenesis	-	-	-	-	-	-	-	-	-	-	6	2,48E-05
GO:0030323 respiratory tube development	-	-	-	-	8	2,00E-04	-	-	-	-	5	4,58E-05
GO:0016540 protein autoprocessing	-	-	-	-	-	-	-	-	-	-	5	6,18E-05
GO:0046777 protein amino acid autophosphorylation	-	-	-	-	-	-	-	-	-	-	5	5,34E-05
GO:0051258 protein polymerization	-	-	-	-	-	-	-	-	-	-	5	1,27E-04
GO:0000226 microtubule cytoskeleton organization and biogenesis	-	-	-	-	-	-	-	-	-	-	6	1,76E-05
GO:0009725 response to hormone stimulus	-	-	-	-	10	1,45E-04	-	-	-	-	6	3,04E-07
GO:0043232 intracellular non-membrane-bound organelle	109	2,68E-04	-	-	-	-	-	-	-	-	-	-
GO:0043229 intracellular organelle	376	2,23E-04	-	-	-	-	-	-	-	-	-	-
GO:0043228 non-membrane-bound organelle	109	2,68E-04	-	-	-	-	-	-	-	-	-	-
GO:0043226 organelle	376	2,34E-04	-	-	-	-	-	-	-	-	-	-
GO:0045111 intermediate filament cytoskeleton	21	4,86E-05	20	5,21E-09	-	-	-	-	-	-	-	-
GO:0044446 intracellular organelle part	185	1,71E-05	101	4,99E-05	-	-	-	-	-	-	-	-
GO:0005615 extracellular space	40	1,31E-04	-	-	-	-	-	-	-	-	-	-
GO:0005882 intermediate filament	21	4,86E-05	20	5,21E-09	-	-	-	-	-	-	-	-
GO:0044424 intracellular part	439	2,24E-04	-	-	-	-	-	-	-	-	-	-
GO:0044422 organelle part	185	2,12E-05	101	5,67E-05	-	-	-	-	-	-	-	-
GO:0006464 protein modification process	106	4,66E-05	-	-	-	-	-	-	-	-	-	-
GO:0005125 cytokine activity	-	-	18	7,87E-06	-	-	-	-	-	-	-	-

*See Table **1** for explanation of acronyms referring to data sets used.
**All p-values were corrected by the Benjamini correction which controls the False Discovery Rate (see Text).

Supplementary Table 3. The Results of Ingenuity Pathway Analysis (http://www.ingenuity.com) Obtained with Genes Overlapping CNVs in Autistic Patients

Lists of genes*

	**CS1_All**		**CS1_Gain**		**CS1_Loss**		**CS2_All**		**CS2_Gain**		**CS2_Loss**	
Functions and Diseases	**B-H P-value**	**# Genes**	**B-H P-value**	**# Genes**	**B-H P-value**	**# Genes**	**B-H P-value**	**# Genes**	**B-H P-value**	**# Genes**	**B-H P-value**	**# Genes**
arrest in G2 phase of tumor cells	-	-	-	-	2,78E-02	3	-	-	-	-	-	-
cytotoxic reaction of embryonic cell lines	5,83E-03	7	3,22E-05	7	1,07E-04	7	7,70E-08	7	-	-	-	-
cytotoxic reaction of cell lines	-	-	4,75E-04	8	1,72E-03	8	9,05E-07	8	-	-	-	-
cytotoxic reaction of eukaryotic cells	4,23E-02	11	3,58E-04	10	1,30E-03	10	1,75E-06	9	-	-	-	-
contact repulsion of axons	-	-	-	-	-	-	-	-	-	-	4,09E-02	2
formation of cytoplasmic aggregates	-	-	-	-	-	-	-	-	-	-	4,09E-02	2
biogenesis of vesicles	-	-	-	-	-	-	-	-	-	-	4,09E-02	2
circadian rhythm of mice	-	-	-	-	-	-	-	-	-	-	4,09E-02	2
collapse of growth cone	-	-	-	-	-	-	-	-	-	-	4,09E-02	3
colony formation of ovarian cancer cell lines	-	-	-	-	-	-	-	-	-	-	4,09E-02	2
chemotaxis of central nervous system cells	-	-	-	-	-	-	-	-	-	-	4,09E-02	2
quantity of endothelial cells	-	-	-	-	-	-	-	-	-	-	4,09E-02	2
quantity of mitochondria	-	-	-	-	-	-	-	-	-	-	4,09E-02	2
targeting of protein	-	-	-	-	-	-	-	-	-	-	4,09E-02	3
accumulation of melano-somes	-	-	-	-	-	-	-	-	-	-	4,09E-02	1
activation of Stat5a response element	-	-	-	-	-	-	-	-	-	-	4,09E-02	1
arrest in G2 phase of endothelial cells	-	-	-	-	-	-	-	-	-	-	4,09E-02	1
atrophy of pancreatic acinus	-	-	-	-	-	-	-	-	-	-	4,09E-02	1
biosynthesis of pyruvic acid	-	-	-	-	-	-	-	-	-	-	4,09E-02	1
branching of N-glycan	-	-	-	-	-	-	-	-	-	-	4,09E-02	1
breakdown of progester-one	-	-	-	-	-	-	-	-	-	-	4,09E-02	1
cardiomyopathy of left ventricle	-	-	-	-	-	-	-	-	-	-	4,09E-02	1

(Suppl Table 3). Contd…..

	CS1_All		CS1_Gain		CS1_Loss		CS2_All		CS2_Gain		CS2_Loss	
Functions and Diseases	**B-H P-value**	**# Genes**	**B-H P-value**	**# Genes**	**B-H P-value**	**# Genes**	**B-H P-value**	**# Genes**	**B-H P-value**	**# Genes**	**B-H P-value**	**# Genes**
chemorepulsion of sympathetic neuron	-	-	-	-	-	-	-	-	-	-	4,09E-02	1
colony formation of mammary tumor cells	-	-	-	-	-	-	-	-	-	-	4,09E-02	1
colony formation of non-small-cell lung cancer cells	-	-	-	-	-	-	-	-	-	-	4,09E-02	1
colony formation of small cell lung cancer cells	-	-	-	-	-	-	-	-	-	-	4,09E-02	1
cystic fibrosis of humans	-	-	-	-	-	-	-	-	-	-	4,09E-02	1
delivery of spermine	-	-	-	-	-	-	-	-	-	-	4,09E-02	1
destabilization of focal adhesions	-	-	-	-	-	-	-	-	-	-	4,09E-02	1
development of accessory nerve	-	-	-	-	-	-	-	-	-	-	4,09E-02	1
development of facial nerve	-	-	-	-	-	-	-	-	-	-	4,09E-02	1
development of fat pad	-	-	-	-	-	-	-	-	-	-	4,09E-02	1
development of glosso-pharyngeal nerve	-	-	-	-	-	-	-	-	-	-	4,09E-02	1
development of mammary lesion	-	-	-	-	-	-	-	-	-	-	4,09E-02	1
development of vagus nerve	-	-	-	-	-	-	-	-	-	-	4,09E-02	1
developmental verbal dyspraxia	-	-	-	-	-	-	-	-	-	-	4,09E-02	1
diameter of left ventricle	-	-	-	-	-	-	-	-	-	-	4,09E-02	1
exit from G0/G1 phase transition of cell lines	-	-	-	-	-	-	-	-	-	-	4,09E-02	1
expansion of basal layer of epidermis	-	-	-	-	-	-	-	-	-	-	4,09E-02	1
expansion of suprabasal layer of epidermis	-	-	-	-	-	-	-	-	-	-	4,09E-02	1
familial advanced sleep phase syndrome	-	-	-	-	-	-	-	-	-	-	4,09E-02	1
filling of crypt	-	-	-	-	-	-	-	-	-	-	4,09E-02	1
filling of lumen	-	-	-	-	-	-	-	-	-	-	4,09E-02	1
formation of calcium stone	-	-	-	-	-	-	-	-	-	-	4,09E-02	1
frequency of breast carcinoma	-	-	-	-	-	-	-	-	-	-	4,09E-02	1
growth of retinal axons	-	-	-	-	-	-	-	-	-	-	4,09E-02	1

(Suppl Table 3). Contd…..

	CS1_All		CS1_Gain		CS1_Loss		CS2_All		CS2_Gain		CS2_Loss	
Functions and Diseases	**B-H P-value**	**# Genes**	**B-H P-value**	**# Genes**	**B-H P-value**	**# Genes**	**B-H P-value**	**# Genes**	**B-H P-value**	**# Genes**	**B-H P-value**	**# Genes**
guidance of olfactory glomeruli	-	-	-	-	-	-	-	-	-	-	4,09E-02	1
hydraulic conductivity of venule	-	-	-	-	-	-	-	-	-	-	4,09E-02	1
hyperproliferation of fibroblasts	-	-	-	-	-	-	-	-	-	-	4,09E-02	1
hypertension of pulmonary artery	-	-	-	-	-	-	-	-	-	-	4,09E-02	1
inflammation of lumen	-	-	-	-	-	-	-	-	-	-	4,09E-02	1
inflammation of submandibular gland	-	-	-	-	-	-	-	-	-	-	4,09E-02	1
innervation of first molar	-	-	-	-	-	-	-	-	-	-	4,09E-02	1
internalization of glycosphingolipid	-	-	-	-	-	-	-	-	-	-	4,09E-02	1
lethal neonatal mitochondrial complex I deficiency	-	-	-	-	-	-	-	-	-	-	4,09E-02	1
metabolism of soleus muscle	-	-	-	-	-	-	-	-	-	-	4,09E-02	1
metastasis of mammary tumor cells	-	-	-	-	-	-	-	-	-	-	4,09E-02	1
morphogenesis of sympathetic trunk	-	-	-	-	-	-	-	-	-	-	4,09E-02	1
morphology of parenchyma	-	-	-	-	-	-	-	-	-	-	4,09E-02	1
obstruction of interlobular duct	-	-	-	-	-	-	-	-	-	-	4,09E-02	1
obstruction of intralobular duct	-	-	-	-	-	-	-	-	-	-	4,09E-02	1
pathfinding of retinal axons	-	-	-	-	-	-	-	-	-	-	4,09E-02	1
polarization of fibroblasts	-	-	-	-	-	-	-	-	-	-	4,09E-02	1
proliferation of non-small-cell lung cancer cells	-	-	-	-	-	-	-	-	-	-	4,09E-02	1
quantity of caveolar membranes	-	-	-	-	-	-	-	-	-	-	4,09E-02	1
quantity of olfactory glomeruli	-	-	-	-	-	-	-	-	-	-	4,09E-02	1
reduction of doxorubicin	-	-	-	-	-	-	-	-	-	-	4,09E-02	1
reduction of vitamin K3	-	-	-	-	-	-	-	-	-	-	4,09E-02	1

(Suppl Table 3). Contd…..

	CS1_All		CS1_Gain		CS1_Loss		CS2_All		CS2_Gain		CS2_Loss	
Functions and Diseases	**B-H P-value**	**# Genes**	**B-H P-value**	**# Genes**	**B-H P-value**	**# Genes**	**B-H P-value**	**# Genes**	**B-H P-value**	**# Genes**	**B-H P-value**	**# Genes**
regulatory volume decrease of epithelial cell lines	-	-	-	-	-	-	-	-	-	-	4,09E-02	1
synthesis of 12(S)-hydroxyeicosatetraenoic acid	-	-	-	-	-	-	-	-	-	-	4,09E-02	1
thickness of interstitial tissue	-	-	-	-	-	-	-	-	-	-	4,09E-02	1
tonicity of blood vessel	-	-	-	-	-	-	-	-	-	-	4,09E-02	1
transcription of ESE box motif	-	-	-	-	-	-	-	-	-	-	4,09E-02	1
transcription of GCF2 binding site	-	-	-	-	-	-	-	-	-	-	4,09E-02	1
volume of pancreatic acinus	-	-	-	-	-	-	-	-	-	-	4,09E-02	1
dilation of organ	-	-	-	-	-	-	-	-	-	-	4,66E-02	2
Canonical Pathways												
Parkinson's Signaling	1,80E-02	6	-	-	-	-	1,50E-03	4	3,60E-03	3	-	-
Coagulation System	-	-	-	-	-	-	-	-	1,70E-02	3	-	-
Activation of IRF by Cytosolic Pattern Recognition Receptors	1,80E-02	11	4,10E-04	10	7,00E-03	9	4,70E-07	10	-	-	-	-
Role of RIG1-like Receptors in Antiviral Innate Immunity	1,80E-02	9	2,80E-04	9	4,30E-03	8	3,20E-06	8	-	-	-	-
Role of Cytokines in Mediating Communication between Immune Cells	1,80E-02	10	4,40E-04	8	4,60E-04	10	1,10E-05	8	-	-	-	-
IL-12 Signaling and Production in Macrophages	-	-	-	-	-	-	1,90E-04	9	-	-	-	-
Systemic Lupus Erythematosus Signaling	-	-	-	-	-	-	4,50E-03	8	-	-	-	-

*see Table **I** for explanation on the content of each CS1 and CS2 gene list.
B-H P values = calculated with the Benjamini-Hochberg correction.

Supplementary Table 4. **It Reports the Regional Expression of 139 Genes from the CS2_All Data Set within the Nervous System***

Gene Symbol	ADRENAL CORTEX	ADRENAL GLAND	AMYGDALA	CEREBELLUM PEDUNCLES	CEREBELLUM	CINGULATE CORTEX	DORSAL ROOT GANGLIA	FETAL BRAIN	HYPOTALAMUS	OCCIPITAL LOBE	OLFACTORY BULB	PARIETAL LOBE	PITUITARY	PONS	PREFRONTAL CORTEX	SPINAL CORD	SUBTHALAMIC NUCLEUS	TEMPORAL LOBE	THALAMUS	WHOLE BRAIN
AASS	•		•		•	•		•					•			•		•		
ACR					•										•				•	
ACSL1	•					•										•	•			•
ADD1	•	•		•	•					•	•		•	•						
AGXT					•				•					•	•					
ALG12								•												•
ARHGAP22					•								•				•			
B4GALT1	•		•		•			•	•			•		•			•		•	•
BAG1		•												•					•	
BRD1																			•	
C10orf72			•		•			•		•	•		•		•		•	•	•	
C15orf2				•	•				•								•			•
C22orf29					•															
C4orf15			•		•					•	•		•		•			•	•	•
C4orf6					•									•						
C4orf8																			•	•
CADPS2		•		•						•	•		•	•						

(Suppl Table 4). Contd…..

Gene Symbol	ADRENAL CORTEX	ADRENAL GLAND	AMYGDALA	CEREBELLUM PEDUNCLES	CEREBELLUM	CINGULATE CORTEX	DORSAL ROOT GANGLIA	FETAL BRAIN	HYPOTALAMUS	OCCIPITAL LOBE	OLFACTORY BULB	PARIETAL LOBE	PITUITARY	PONS	PREFRONTAL CORTEX	SPINAL CORD	SUBTHALAMIC NUCLEUS	TEMPORAL LOBE	THALAMUS	WHOLE BRAIN
CAPZA2												•								•
CASP3					•															•
CAV1	•				•	•		•								•		•		
CAV2	•				•	•		•	•			•		•		•	•	•		
CD83			•	•						•										
CDKN2A			•		•		•							•			•			
CDKN2B					•									•						
CFTR			•		•	•		•	•	•		•		•		•		•	•	
CHAT					•									•						
CLTCL1			•		•									•					•	
CRKL					•															•
CRMP1		•		•			•			•	•	•	•	•	•		•			
CTBP1	•	•		•			•	•			•		•	•						•
DAP	•				•			•				•								•
DGCR14	•	•	•	•	•				•			•		•					•	•
DGKQ					•									•						
DKFZP434L187			•		•							•								
F11					•			•							•			•		

(Suppl Table 4). Contd…..

Gene Symbol	ADRENAL CORTEX	ADRENAL GLAND	AMYGDALA	CEREBELLUM PEDUNCLES	CEREBELLUM	CINGULATE CORTEX	DORSAL ROOT GANGLIA	FETAL BRAIN	HYPOTALAMUS	OCCIPITAL LOBE	OLFACTORY BULB	PARIETAL LOBE	PITUITARY	PONS	PREFRONTAL CORTEX	SPINAL CORD	SUBTHALAMIC NUCLEUS	TEMPORAL LOBE	THALAMUS	WHOLE BRAIN
FARP2					•														•	
FLJ44385			•							•	•		•		•			•	•	
GABRA5		•	•		•		•	•		•	•		•		•					
GABRB3					•						•			•	•					
GPC1	•				•	•	•		•	•	•			•				•	•	
GRK4					•			•											•	
HDLBP	•				•							•		•					•	
HGFAC					•															
IDUA					•							•		•						•
IFNA10					•									•						
IFNA14					•															
IFNA16					•									•						
IFNA17					•									•						
IFNA21					•													•		
IFNA6			•		•				•			•	•		•			•		
IFNA7					•			•												
IFNB1					•				•									•		
IFNW1					•											•				

(Suppl Table 4). Contd…..

Gene Symbol	ADRENAL CORTEX	ADRENAL GLAND	AMYGDALA	CEREBELLUM PEDUNCLES	CEREBELLUM	CINGULATE CORTEX	DORSAL ROOT GANGLIA	FETAL BRAIN	HYPOTALAMUS	OCCIPITAL LOBE	OLFACTORY BULB	PARIETAL LOBE	PITUITARY	PONS	PREFRONTAL CORTEX	SPINAL CORD	SUBTHALAMIC NUCLEUS	TEMPORAL LOBE	THALAMUS	WHOLE BRAIN
ILKAP		•																		
INSL4					•				•											
INSL6					•									•						
IPW				•			•	•		•			•	•	•		•			
IRX4			•		•				•				•	•		•		•		
KLHL9					•	•								•	•	•	•	•		•
KLKB1			•		•									•						
LETM1					•				•					•						
LOC285484			•							•	•		•		•			•	•	
Lrp2bp					•															
LRRFIP1			•		•							•						•		•
LSM8			•							•	•		•		•			•	•	•
MAEA		•																		•
MAGEL2		•			•									•						
MAPK12			•					•	•		•		•	•	•			•		
MAPK8				•	•							•	•	•			•		•	
MCM10					•															
MLC1		•		•			•			•	•				•		•			

(Suppl Table 4). Contd…..

Gene Symbol	ADRENAL CORTEX	ADRENAL GLAND	AMYGDALA	CEREBELLUM PEDUNCLES	CEREBELLUM	CINGULATE CORTEX	DORSAL ROOT GANGLIA	FETAL BRAIN	HYPOTALAMUS	OCCIPITAL LOBE	OLFACTORY BULB	PARIETAL LOBE	PITUITARY	PONS	PREFRONTAL CORTEX	SPINAL CORD	SUBTHALAMIC NUCLEUS	TEMPORAL LOBE	THALAMUS	WHOLE BRAIN
MLF1IP					•													•	•	
MLLT3		•			•	•		•		•				•						
MLPH								•	•			•		•						
MSX1	•	•			•			•				•		•			•	•		
MTAP			•	•	•	•		•	•					•		•		•		•
MTERFD2					•				•								•			
MTRR					•					•	•		•	•	•		•			
MXD4	•	•		•	•				•					•			•			
NDN	•	•					•				•			•						
NDUFA10	•	•							•											
NDUFS6									•											
NFX1					•													•		
NMB	•				•	•		•				•		•			•			
OCA2					•				•				•	•						
OGDHL		•		•			•	•		•	•		•	•	•					
OPTN	•	•					•			•	•	•	•				•		•	
PASK					•		•					•							•	•
PER2		•		•	•							•		•					•	

(Suppl Table 4). Contd…..

Gene Symbol	ADRENAL CORTEX	ADRENAL GLAND	AMYGDALA	CEREBELLUM PEDUNCLES	CEREBELLUM	CINGULATE CORTEX	DORSAL ROOT GANGLIA	FETAL BRAIN	HYPOTALAMUS	OCCIPITAL LOBE	OLFACTORY BULB	PARIETAL LOBE	PITUITARY	PONS	PREFRONTAL CORTEX	SPINAL CORD	SUBTHALAMIC NUCLEUS	TEMPORAL LOBE	THALAMUS	WHOLE BRAIN
PGBD3					•									•						
PPP1R7		•			•		•												•	
PTPRZ1		•		•			•			•	•		•		•		•			
RAB17					•			•				•		•						
RABL2B		•			•		•										•		•	
RAMP1		•			•		•		•		•				•		•			
RB1CC1					•					•	•									•
RGS12	•	•	•		•	•			•	•	•	•	•	•	•	•		•	•	
RNF4		•																		•
RPS6					•	•						•		•				•		•
SCAND2			•		•	•			•	•			•		•			•		
SCLY		•			•							•		•						
SDF2L1												•		•						
SEMA3A	•				•				•					•				•		
SEMA3D			•		•	•		•								•				
SEMA5A	•				•	•							•				•			
SEPT2	•				•			•						•			•			
SERPIND1					•	•											•			

(Suppl Table 4). Contd…..

Gene Symbol	ADRENAL CORTEX	ADRENAL GLAND	AMYGDALA	CEREBELLUM PEDUNCLES	CEREBELLUM	CINGULATE CORTEX	DORSAL ROOT GANGLIA	FETAL BRAIN	HYPOTALAMUS	OCCIPITAL LOBE	OLFACTORY BULB	PARIETAL LOBE	PITUITARY	PONS	PREFRONTAL CORTEX	SPINAL CORD	SUBTHALAMIC NUCLEUS	TEMPORAL LOBE	THALAMUS	WHOLE BRAIN
SH3BP2					•									•						•
SLC18A3					•									•				•		
SLC24A2						•								•	•					
SLC25A1	•								•											
SLC25A4		•							•		•		•	•				•		
SLC26A1					•							•		•			•	•		
SLC28A1															•			•		
SLC4A10			•		•	•			•						•			•		
SMU1					•															
SNAP29																			•	
SNURF		•		•	•		•	•		•	•		•	•	•		•	•		
SOX12					•			•												
SPINK4			•						•					•				•		
SPON2	•								•			•						•		•
SRD5A1		•			•		•					•								
ST18				•	•		•			•	•	•	•	•	•	•	•			
ST7				•	•	•						•		•						
STK25		•																		

(Suppl Table 4). Contd…..

Gene Symbol	ADRENAL CORTEX	ADRENAL GLAND	AMYGDALA	CEREBELLUM PEDUNCLES	CEREBELLUM	CINGULATE CORTEX	DORSAL ROOT GANGLIA	FETAL BRAIN	HYPOTALAMUS	OCCIPITAL LOBE	OLFACTORY BULB	PARIETAL LOBE	PITUITARY	PONS	PREFRONTAL CORTEX	SPINAL CORD	SUBTHALAMIC NUCLEUS	TEMPORAL LOBE	THALAMUS	WHOLE BRAIN
SUCLG2	•				•			•				•		•						•
TAS2R1					•									•						
TES					•														•	•
TETRAN								•												
TFEC					•	•										•				•
TLR3			•		•					•	•		•	•	•			•	•	
TNIP2																				•
TRAF3IP1					•									•		•				
TRIB3					•							•								
TSSK2			•	•	•				•			•						•	•	•
WHSC1					•			•											•	
WHSC2					•									•						
WNT2					•					•				•						
ZBED4					•									•	•					

*The data reported in this Table were retrieved using the "Tissue expression" function of the D.A.V.I.D. software and refer to information stored in the Genome Informatics Applications & Datasets (GNF_U133A) database from Genomics Institute of the Novartis Research Foundation.

Supplementary Table 5. It Reports Detailed Information on the Nervous System Related Genes (i.e., 43 Genes Listed in Table 3) and in Particular (i) the Different GO Functional Categories Associated to Each Gene, (ii) the Number of the Splicing Isoforms for the same Genes when Present*

Gene	Gain/Loss	GO Biological Process	GO Molecular Function	Alternative Splicing Isoforms
ADAM10	Loss	in utero embryonic development; protein amino acid phosphorylation; proteolysis; negative regulation of cell adhesion; Notch signaling pathway; integrin-mediated signaling pathway; cell-cell signaling; positive regulation of cell proliferation.	metalloendopeptidase activity; integrin binding peptidase; activity zinc ion binding SH3; domain binding protein kinase; binding protein; omodimerization activity; metal ion binding.	7
ASZ1	Loss	neuron migration; negative regulation of endothelial cell proliferation; synaptic transmission; brain development; lactation; sperm motility; transepithelial chloride transport; lung development; positive regulation of microtubule polymerization; vasodilatation.	protein tyrosine kinase activity; signal transducer activity; kinase binding; syntaxin binding; nitric-oxide synthase binding.	4
BMP15	Gain	female gamete generation.	cytokine activity; growth factor activity.	-
C4orf6	Gain	nervous system development.	-	-
CADPS2	Loss	Exocytosis; protein transport.	calcium ion binding; lipid binding.	3
CASP3	Gain	B cell homeostasis; release of cytochrome c from mitochondria; DNA fragmentation during apoptosis; proteolysis; induction of apoptosis; response to DNA damage stimulus; heart development; sensory perception of sound; induction of apoptosis via death domain.	cysteine-type endopeptidase activity; cyclin-dependent protein kinase inhibitor activity; protein binding; peptidase activity.	4
CAV1	Loss	inactivation of MAPK activity; vasculogenesis; response to hypoxia; negative regulation of endothelial cell proliferation; triglyceride metabolic process; calcium ion transport; cellular calcium ion homeostasis; endocytosis; regulation of smooth muscle contraction.	structural molecule activity; protein binding; cholesterol binding; peptidase activator activity; nitric-oxide synthase binding.	6
CAV2	Loss	vesicle fusion; vesicle organization; vesicle docking.	protein binding; protein homodimerization activity.	4
CHAT	Gain	neuromuscular synaptic transmission; muscle development; establishment of synaptic specificity at neuromuscular junction; rhythmic behavior; adult walking behavior; dendrite development; neuron differentiation; neurotransmitter biosynthetic process.	choline O-acetyltransferase activity; acyltransferase activity; transferase activity.	6
CRKL	Gain	blood vessel development intracellular; signaling cascade JNK cascade Ras; protein signal transduction pattern; specification process.	protein tyrosine kinase activity; SH3/SH2 adaptor activity; protein binding.	-
CRMP1	Gain	nucleobase, nucleoside, nucleotide and nucleic acid metabolic process; nervous system development.	dihydropyrimidinase activity; protein binding; hydrolase activity, acting on carbon-nitrogen (but not peptide) bonds.	8
CTTNBP2	Loss	neuron migration; negative regulation of endothelial cell proliferation; synaptic transmission; brain development; lactation; sperm motility; transepithelial chloride transport; lung development; positive regulation of microtubule polymerization; vasodilatation.	protein tyrosine kinase activity; kinase binding; syntaxin binding; nitric-oxide synthase binding.	8
DGCR14	Gain	mRNA processing; nervous system development; RNA splicing.	molecular function.	7
EPHA7	Gain	protein amino acid phosphorylation; transmembrane receptor protein tyrosine kinase signaling pathway.	nucleotide binding; receptor activity; ephrin receptor; activity protein; binding ATP binding.	4

(Suppl Table 5). Contd…..

Gene	Gain/Loss	GO Biological Process	GO Molecular Function	Alternative Splicing Isoforms
FARP2	Loss	neuron remodeling; Rac protein signal transduction; regulation of Rho protein signal transduction .	guanyl-nucleotide exchange factor activity; Rho guanyl-nucleotide exchange factor activity; binding; cytoskeletal protein binding.	9
FOXP2	Loss	transcription; regulation of transcription, DNA-dependent; caudate nucleus development; putamen development	transcription factor activity; zinc ion binding; protein homodimerization activity; sequence-specific DNA binding; metal ion binding.	8
FZD6	Gain	establishment of planar polarity; neural tube closure; G-protein coupled receptor protein signaling pathway; multicellular organismal development; Wnt receptor signaling pathway; inner ear morphogenesis.	non-G-protein coupled 7TM receptor activity; G-protein coupled receptor activity; Wnt receptor activity.	3
GPR37	Loss	signal transduction; G-protein coupled receptor protein signaling pathway.	receptor activity; G-protein coupled receptor activity.	-
HES6	Loss	regulation of transcription, DNA-dependent; multicellular organismal development; cell differentiation.	transcription factor activity; transcription cofactor activity.	3
MAPK8	Gain	nucleotide binding; protein serine/threonine kinase activity; JUN kinase activity; MAP kinase activity; protein binding; ATP binding; transferase activity.	nucleotide binding; protein serine/threonine kinase activity; JUN kinase activity; MAP kinase activity; protein binding; ATP binding; transferase activity.	6
MAPK11	Loss	signal transduction; G-protein coupled receptor protein signaling pathway.	receptor activity; G-protein coupled receptor activity.	-
MAPK12	Loss	DNA damage induced protein phosphorylation; cell cycle; cell cycle arrest; intracellular signaling cascade; Ras protein signal transduction.	nucleotide binding; magnesium ion binding; protein serine/threonine kinase activity; MAP kinase activity; protein binding.	13
MLC1	Loss	ion transport; biological process.	molecular function; ion channel activity.	20
MSX1	Gain	negative regulation of transcription from RNA polymerase II promoter; in utero embryonic development; heart morphogenesis; multicellular organismal development; muscle development; negative regulation of cell proliferation; embryonic limb morphogenesis.	transcription factor activity; protein binding; transcription repressor activity; sequence-specific DNA binding.	-
NDN	Gain	neuron migration; transcription; regulation of transcription, DNA-dependent; axonal fasciculation; central nervous system development; respiratory gaseous exchange; negative regulation of cell proliferation; glial cell migration; sensory perception of pain.	DNA binding; protein binding; gamma-tubulin binding.	-
OPN1SW	Loss	signal transduction; G-protein coupled receptor protein signaling pathway; visual perception; phototransduction; protein-chromophore linkage; response to stimulus.	G-protein coupled receptor activity; photoreceptor activity.	-
PDGFA	Loss	cell activation; cell-cell signaling; embryonic development; negative regulation of phosphatidylinositol biosynthetic process; negative regulation of platelet activation; regulation of smooth muscle cell migration; positive regulation of cell migration.	platelet-derived growth factor receptor binding; collagen binding; growth factor activity; protein homodimerization activity; cell surface binding; protein heterodimerization activity; platelet-derived growth factor binding.	2
PER2	Loss	transcription; regulation of transcription, DNA-dependent; signal transduction; circadian rhythm.	signal transducer activity; protein binding.	2
PIK3C3	Gain	protein amino acid phosphorylation; phosphoinositide phosphorylation; phosphoinositide-mediated signalling.	nucleotide binding; inositol or phosphatidylinositol kinase activity; protein kinase activity; protein binding; ATP binding; 1-phosphatidylinositol-3-kinase activity; transferase activity; manganese ion binding.	11

(Suppl Table 5). Contd…..

Gene	Gain/Loss	GO Biological Process	GO Molecular Function	Alternative Splicing Isoforms
PLXNB2	Loss	signal transduction; multicellular organismal development; positive regulation of axonogenesis.	receptor activity; protein binding.	30
PRKAR1B	Loss	regulation of protein amino acid phosphorylation; signal transduction; hormone-mediated signalling.	nucleotide binding; cAMP-dependent protein kinase regulator activity; cAMP binding.	-
PTPRZ1	Loss	protein amino acid dephosphorylation; central nervous system development.	transmembrane receptor protein tyrosine phosphatase activity; protein tyrosine/threonine phosphatase activity; hydrolase activity.	2
RAC3	Gain	small GTPase mediated signal transduction; cell projection assembly; actin cytoskeleton organization; neurite development; neuromuscular process controlling balance.	nucleotide binding; GTPase activity; protein binding; GTP binding.	5
SEMA3A	Loss	regulation of heart rate; multicellular organismal development; nervous system development; axon guidance; axonal fasciculation; cell differentiation; negative regulation of axon extension involved in axon guidance; negative chemotaxis.	receptor activity; chemorepellent activity.	-
SEMA3D	Loss	multicellular organismal development; nervous system development; cell differentiation.	receptor activity.	-
SEMA5A	Loss	patterning of blood vessels; cell adhesion; cell-cell signaling; multicellular organismal development; nervous system development; axon guidance; cell differentiation.	receptor activity; axon guidance receptor activity.	-
SEMA7A	Loss	immune response; multicellular organismal development; nervous system development; cell differentiation.	receptor activity; protein binding.	-
SEPT5	Loss	cytokinesis; cell cycle; synaptic vesicle targeting; positive regulation of exocytosis.	nucleotide binding; GTPase activity; structural molecule activity; protein binding; GTP binding.	9
SMO	Loss	vasculogenesis; osteoblast differentiation; neural crest cell migration; positive regulation of neuroblast proliferation; heart morphogenesis; G-protein coupled receptor protein signaling pathway; determination of left/right symmetry; pattern specification.	non-G-protein coupled 7TM receptor activity; G-protein coupled receptor activity; protein binding.	3
SPON2	Gain	immune response; cell adhesion; axon guidance.	protein binding.	7
WASL	Loss	transcription; regulation of transcription, DNA-dependent; protein complex assembly; cell motion; actin polymerization or depolymerization; response to bacterium.	actin binding; small GTPase regulator activity; protein binding.	-
WNT16	Loss	Wnt receptor signaling pathway, calcium modulating pathway; cell-cell signaling; multicellular organismal development.	signal transducer activity; extracellular matrix structural constituent.	2
WNT2	Loss	neuron migration; negative regulation of endothelial cell proliferation; Wnt receptor signaling pathway, calcium modulating pathway; synaptic transmission; multicellular organismal development; brain development; lactation; sperm motility.	protein tyrosine kinase activity; signal transducer activity; extracellular matrix structural constituent; kinase binding; syntaxin binding; nitric-oxide synthase binding.	4

*The 43 genes in this Table are the same reported in Table **3** (see Results for additional information).

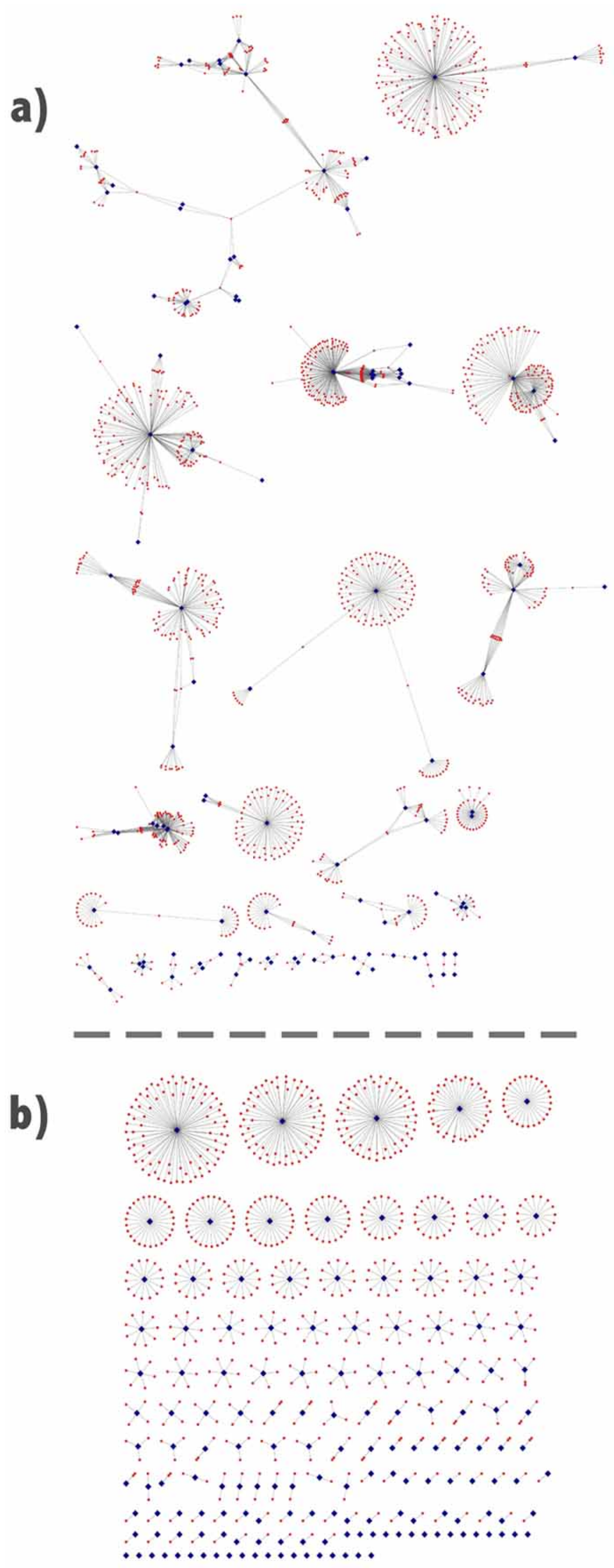

Supplementary Fig. (1). Bipartite networks drawn by Cytoscape (http://www.cytoscape.org) showing the genes deleted or duplicated only in a single patient (**b**) or in two or more patients (**a**). Red circles: genes; Blue circles: patients. Genes shared by two or more patients are indicated as red circles connected to blue circles by two or more lines. Overall this analysis showed that 113 out of 233 (49 % ca.) patients share at least one gene with at least another patient and that the total number of "shared" genes among patients is 514.

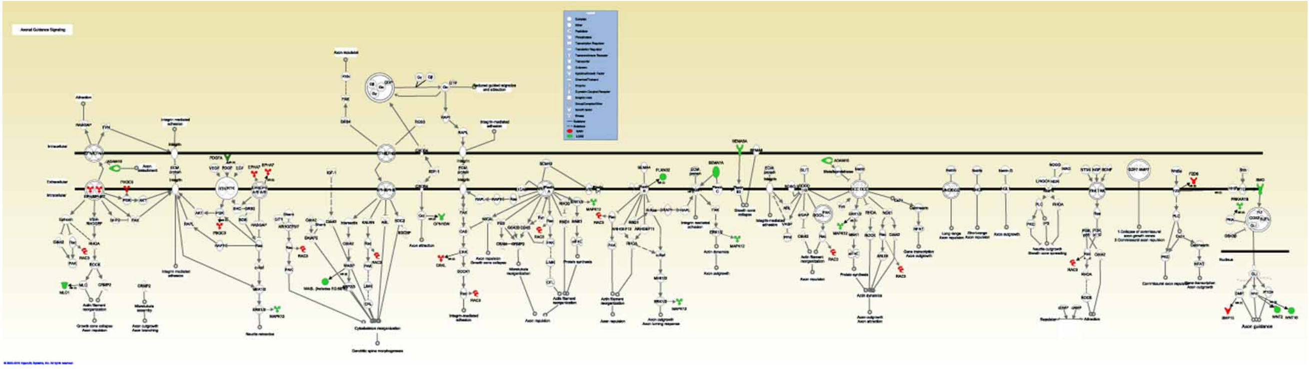

Supplementary Fig. (2). The I.P.A. "axon guidance signalling" pathway with the indication (see symbols' legend on the figure) of the proteins encoded by the genes deleted or duplicated in autistic patients. These proteins appear to act at different levels of the same or a different signalling cascade involved in this pathway.
